# Knowledge, attitude and practice towards dengue fever prevention and treatment among health care workers in Somalia: a cross-sectional study

**DOI:** 10.1186/s12913-025-12820-8

**Published:** 2025-05-09

**Authors:** Fartun Abdullahi Hassan Orey, Salah Ahmed Nur, Suleiman Elmi Ahmed, Bashiru Garba, Mohamed Mustaf Ahmed, Jaweriya Bashir Ahmed, Hamdi Mohamed Isse, Hodo Aideed Asowe, Jihaan Hassan, Najib Isse Dirie, Mohamed Hussein Adam, Jamal Hassan Mohamoud

**Affiliations:** 1 Department of Pediatric and Child Health, Dr. Sumait Hospital, Faculty of Medicine and Health Science, SIMAD University, Mogadishu, Somalia; 2 Faculty of Medicine and Health Science, SIMAD University, Mogadishu, Somalia; 3 Department of Radiology, Faculty of Medicine and Health Sciences, SIMAD University, Mogadishu, Somalia; 4 Department of Nursing and Midwifery Science, Faculty of Medicine and Health Science, SIMAD University, Mogadishu, Somalia; 5 Department of Urology, Dr. Sumait Hospital, Faculty of Medicine and Health Science, SIMAD University, Mogadishu, Somalia; 6 Department of Public Health, Faculty of Medicine and Health Science, SIMAD University, Mogadishu, Somalia; 7 SIMAD Institute for Global Health (SIGHt), SIMAD University, Mogadishu, Somalia

**Keywords:** Dengue fever, Vector-borne disease, Somalia, Healthcare workers, Infectious disease

## Abstract

**Background:**

Dengue fever (DF) has become a public health concern due to its rapid spread worldwide. In 2022, dengue outbreak cases were reported in Mogadishu. Therefore, evaluating healthcare ’workers’ knowledge, attitudes, and practices will substantially impact the prevention and treatment of DF patients. This study aimed to healthcare workers’ knowledge, attitudes, and practices concerning DF.

**Method:**

A descriptive cross-sectional design was conducted from March 2023 to July 2023. The study participants were 384 healthcare workers from multiple federal states in Somalia. A structured questionnaire was disseminated online, especially via WhatsApp, Messenger, and email. Data was cleaned, coded, and analysed using R programming software (version 4.4.0).

**Result:**

The 384 healthcare workers conducted across Somalia demonstrated 66.66% poor knowledge (16.2 ± 4.7), 70.9% positive attitudes (mean score 45.3 ± 4.2), and 61.6% poor practices (mean score 11.7 ± 4.7). Correlation analysis showed that knowledge showed a moderate correlation with practices (*r* = 0.49) and a weak correlation with attitudes (*r* = 0.30), while attitudes and practices demonstrated a weak correlation (*r* = 0.25).

**Conclusion:**

This study identified critical gaps in knowledge and practice regarding DF among healthcare workers in Somalia. Targeted training programs on critical knowledge among health workers, community engagement strategies, and behavioural interventions will help prevent and treat DF in Somalia.

## Background

Dengue fever (DF) is a re-emerging disease that poses a significant global public health challenge due to its rapid increase in incidence worldwide [1]. Commonly referred to as “breakbone fever,” DF is caused by the dengue virus and transmitted to humans through the bites of infected *Aedes aegypti* and *Aedes albopictus* mosquitoes [2]. Clinically, the disease can range from asymptomatic to symptomatic, characterised by fever lasting 5 to 7 days, accompanied by symptoms such as headache, retro-orbital pain, myalgia, arthralgia, and rash. Severe complications, including hemorrhagic manifestations, leukopenia, and organ impairment, may arise in a small proportion of cases [3–5]. According to the World Health Organisation (WHO), the incidence of dengue has increased over eightfold between 2000 and 2010, rising from 500,000 cases to over 5 million annually, with significant underreporting and misdiagnosis likely exacerbating the burden [2]. Each year, an estimated 500,000 individuals develop severe dengue, with a fatality rate of approximately 5%, resulting in nearly 20,000 deaths globally [6].

DF remains underdiagnosed and underreported in Africa despite documented outbreaks in countries such as Somalia, Mauritania, Ethiopia, Kenya, and Senegal [7–11]. In particular, Somalia has a notable history of dengue outbreaks. During 1986–1987, a malaria-like illness characterised by fever, back and joint pain, sweating, and headaches was reported in the Dam Camp, a refugee settlement near Hargeisa [12]. A retrospective study conducted in 1994 among U.S. troops in Somalia revealed that 45% of febrile cases were attributable to DF [13]. More recently, in June 2011, the African Union Mission in Somalia (AMISOM) peacekeepers in Mogadishu experienced an outbreak of an acute febrile illness, resulting in three fatalities. Subsequent blood tests confirmed the presence of the dengue virus among the affected soldiers [7]. In 2022, a dengue outbreak was reported in Banadir Region, with the first confirmed case identified on September 11. This was followed by an active case investigation in October after a reported fatality and two confirmed cases in the region [8]. A study conducted in Mogadishu revealed that over 10% of participants tested positive for DF, underscoring its growing impact on public health [8].

Healthcare workers, including physicians, nurses, and laboratory technicians, play a critical role in diagnosing, treating, and reporting dengue cases. Their knowledge, attitudes, and practices significantly influenced the effectiveness of dengue prevention and control efforts. In Somalia, where underreporting and misdiagnosis of DF are common, understanding the perspectives of healthcare workers is essential for designing effective control strategies. This study aimed to evaluate healthcare workers’ knowledge, attitudes, and practices regarding DF. By identifying gaps in knowledge and areas for improvement, this study seeks to provide evidence-based recommendations to enhance healthcare professionals’ capacity effectively manage and control dengue outbreaks.

## Methodology

### Study design and setting

This study utilized a cross-sectional design to assess knowledge, attitudes, and practices (KAP) regarding DF among healthcare workers residing in different regions of Somalia. It was conducted from 22nd March 2023 to 4 th July 2023.

### Study population and sampling

The target population included healthcare workers in the selected regions (Somaliland, Puntland, Galmudug, Hir-Shabelle, South-west, Jubaland and Benadir region) in Somalia who were available during data collection.

The sample size was determined using the Kish and Leslie formula as follows [14]:

$$\:n=\frac{{Z}^{2}p(1-p)}{{e}^{2}}$$

n = Sample Size

Z Confidence level at 95% (standard value of 1.96)

P Estimated Prevalence of knowledge about DF prevention 0.5

e = Marginal error

where n represents the desired sample size, Z is 1.96 (CI 95%), and e is the margin of error set at 5% (0.05). The estimated prevalence of knowledge of DF was set at 0.5. The calculated sample size was 384.

Convenience sampling was employed to reach healthcare workers for this study. Healthcare workers were invited to participate through direct outreach at hospitals in Mogadishu, and personal networks such as WhatsApp were used for those residing in different regions. Measures were also taken to identify and exclude duplicate responses and to enhance data quality by reducing potential redundancies within the convenience sampling framework.

### Data collection and measures

Data were gathered using a structured, self-administered questionnaire adopted and modified from a previous study using Google Forms [15]. After the modification to the adopted questionnaire, a further assessment to validate its contents and consistency was done with the help of a panel of three experts (a nurse, a medical doctor, and a laboratory technologist), before the questionnaire was pilot tested on 30 respondents recruited randomly from some selected hospitals. The instrument was organised into five distinct sections to capture relevant information comprehensively. Section 1 focused on sociodemographic data, including age, gender, professional role, and area of residence, providing a background for contextualising healthcare workers’ responses. Section 2 assessed healthcare workers’ knowledge of DF, specifically evaluating their understanding of symptoms, treatment protocols, and transmission pathways. Section 3 explores their perceptions and beliefs about DF severity, preventability, control measures, and the perceived roles of individuals and health professionals in dengue prevention and treatment. Section 4 evaluates preventive practices and analyses the extent to which healthcare workers personally engage in DF prevention, explicitly focusing on actions that mitigate mosquito breeding and reduce exposure to mosquito bites. Finally, Sect. 5 investigated treatment-seeking behaviour, focusing on actions taken by healthcare workers if they experience symptoms potentially related to DF.

The questionnaire was disseminated online, specifically WhatsApp and email, to ensure broad accessibility among healthcare professionals. Trained data collectors closely monitored the data collection process and provided follow-up support to enhance the response rate. Additionally, they facilitated communication to address any queries, clarifications, or technical difficulties, ensuring data accuracy and completeness in responses.

### Data analysis and interpretation

Data was cleaned, coded, and using R programming software (version 4.4.0). Descriptive statistics were used to summarise the data. Categorical data are presented as frequencies and percentages, whereas continuous data are presented as means and standard deviations. To ensure confidentiality, no personal identifiers were collected. In addition, there were no missing data points in the final dataset. The healthcare professionals’ knowledge, attitudes and practices were evaluated based on their responses to a structured questionnaire. Knowledge assessment consisted of multiple true/false statements, where correct answers were scored as one and incorrect answers as 0. Using ’Bloom’s original cut-off point, knowledge scores were sdichotomised into two categories: poor knowledge (≤ 60% of the total score, corresponding to 0–18 points) and good knowledge (> 60% of the total score, corresponding to 18–30 points) [16].

Attitude statements were evaluated using a 5-point Likert scale ranging from “strongly agree” (5 points) to “strongly disagree” (1 point). For reverse-coded statements, the scoring was inverted, with “strongly disagree” receiving 5 points and “strongly agree” receiving 1 point. The total attitude scores were categorised as negative (≤ 60% of the total score, corresponding to 14–42 points) and positive (> 60% of the total score, corresponding to 43–70 points). Practice assessment included 23 yes/no statements, with “yes” responses (indicating correct practice) scored as one and “no” responses scored as 0. Similar to the knowledge and attitude scoring, practice scores were classified as poor practice (≤ 60% of the total score, corresponding to 0–14 points) and good practice (> 60% of the total score, corresponding to 14–23 points). The relationships between the knowledge, attitude, and practice scores were analysed using Pearson correlation analysis. Statistical significance was set at *p* < 0.05.

## Results

### Sociodemographic characteristics

**Fig. 1:**
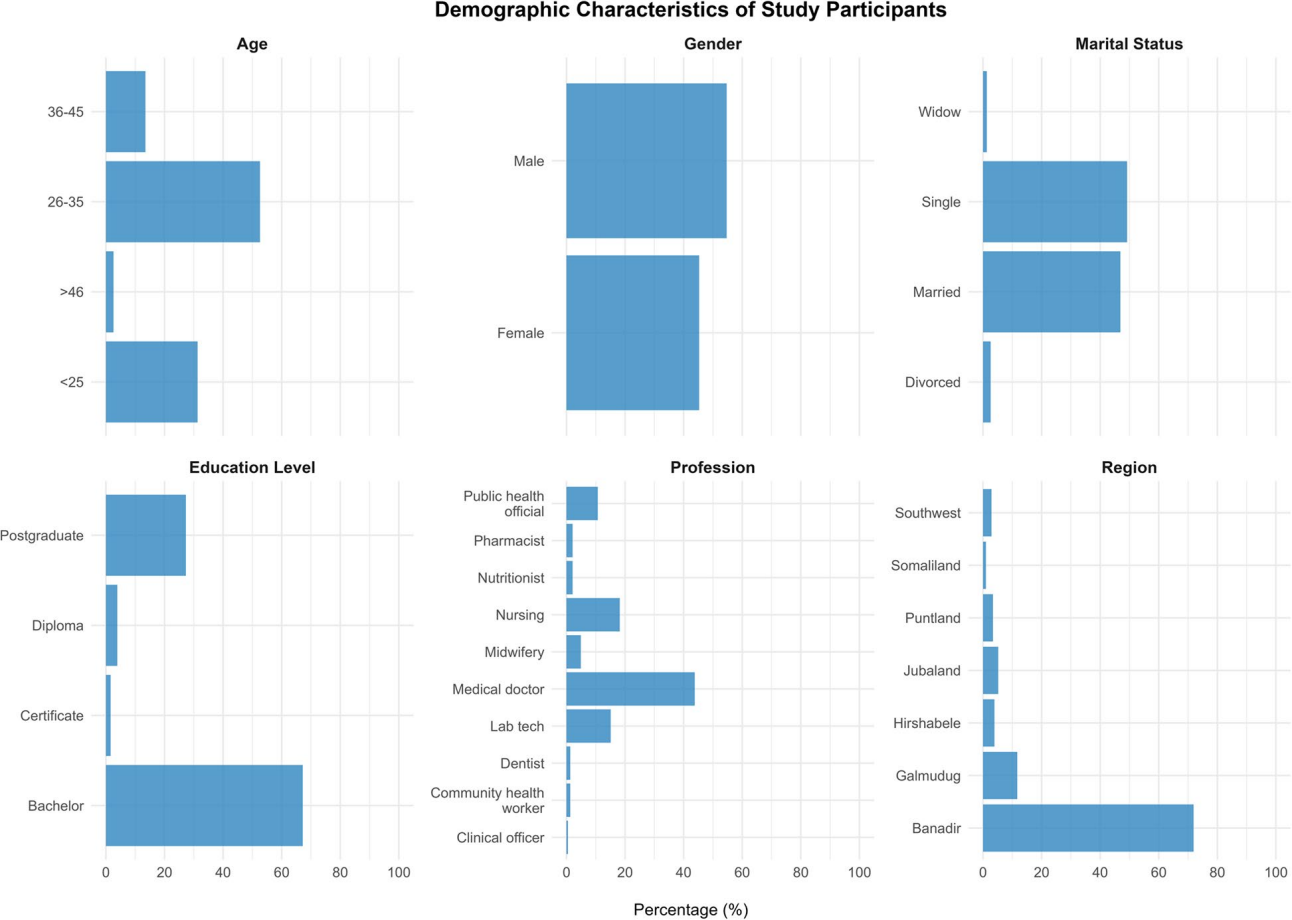
Demographic characteristics of healthcare workers participating in the knowledge, attitude and practice study on dengue disease in Somalia

The study included 384 healthcare professionals across Somalia, revealing a predominantly young workforce, with 52.6% aged 26–35 years and 31.3% under 25 years, indicating a relatively nascent healthcare system (Fig. 1). The gender distribution showed a moderate male predominance (54.7% vs. 45.3%), suggesting a relatively balanced gender representation among the participants. Marital status analysis revealed that nearly half (49.2%) were single, closely followed by married professionals (46.9%), with a minimal representation of divorced (2.6%) and widowed (1.3%) individuals. Educational attainment analysis demonstrated that most healthcare professionals held a degree (67.2%), and 27.3% had postgraduate qualifications (Fig. 1). The professional composition revealed a physician-heavy workforce (43.8%), followed by nurses (18.2%), and laboratory technicians (15.1%). The regional distribution showed significant centralisation in Banadir (71.9%), followed by Galmudug (11.7%), highlighting geographical disparities in the distribution of healthcare professionals.

### Knowledge assessment

Knowledge assessment revealed concerning patterns, with 66.66% demonstrating poor knowledge (≤ 60% correct responses) and a mean score of 16.2 ± 4.7 (Table 1). Symptom recognition showed an apparent dichotomy: high awareness of common symptoms (fever, 96.88%; chills, 84.64%; headache, 81.25%) but poor recognition of critical warning signs (pain behind eyes, 44.27%; bleeding manifestations, 34.64%). Disease transmission knowledge showed moderate understanding, with 70.05% correctly identifying mosquitoes as vectors, and 64.06% specifically recognising Aedes mosquitoes. Vector behaviour knowledge revealed gaps, with only 17.19% correctly identifying peak activity periods, despite 71.88% knowing that mosquitoes were always active. Management knowledge was strongest for basic interventions (paracetamol use 89.06%, rest importance 71.35%) but showed deficiencies in critical aspects, such as hydration awareness (60.94%).

**Table 1:** Knowledge on symptoms, transmission, and management of DF among healthcare workers in Somalia

Knowledge level	Frequency *n* (%)	Mean ± SD
Poor Knowledge (≤ 60%) (0–18 scores)	256 (66.66)	16.2 ± 4.7
Good Knowledge (> 60%) (18–30 scores)	128 (33.33)	
Total	384 (100)	

**Category**	**Item**	**Correct n (%)**
Signs and Symptoms		
Common Symptoms	Fever	372 (96.88)
	Chills	325 (84.64)
	Headache	312 (81.25)
	Muscle pain	248 (64.58)
	Joint pain	240 (62.50)
	Nausea and vomiting	256 (66.67)
Warning Signs	Pain behind eyes	170 (44.27)
	Painful Backbone	173 (45.05)
	Stomach pain	122 (31.77)
	Bleeding of nose, gums and skin	133 (34.64)
	Skin rash	180 (46.88)
	Swollen glands	151 (39.32)
	Restlessness	247 (64.32)
	Pale or cold to touch	100 (26.04)
	Respiratory disturbances	176 (45.83)
	Circulatory disturbances	185 (48.18)
Disease Transmission	Mosquitoes transmit dengue	269 (70.05)
	Aedes mosquitoes transmit dengue	246 (64.06)
	Disease is not contagious	150 (39.06)
Vector Behavior		
Timing	Mosquitoes active at all times	276 (71.88)
	Evening and midnight	159 (41.41)
	Dawn and late afternoon	66 (17.19)
	Morning and noon	36 (9.38)
Breeding Sites	Discarded containers breed mosquitoes	337 (87.76)
Seasonal Pattern	Rainy season correlation	190 (49.48)
Management	Paracetamol use	342 (89.06)
	Rest importance	274 (71.35)
	Adequate hydration	234 (60.94)
	Specific treatments exist	203 (52.86%)
	Herbal remedies effective	40 (10.42)

### Attitude evaluation

The attitude assessment revealed that 70.9% demonstrated positive attitudes (mean score 45.3 ± 4.2) (Table 2). Professional responsibility awareness was high, with 86.72% acknowledging their role in prevention (41.67% strongly agreed and 45.05% agreed). However, significant misconceptions persisted: 70.05% believed that mosquito elimination was the sole prevention method (38.02% strongly agreed, 32.03% agreed), and 59.9% incorrectly believed eradication was the responsibility of health professionals and volunteers only (25.26% strongly agreed, 34.64% agreed). Community engagement attitudes showed mixed results, with 69.79% supporting breeding ground monitoring (40.62% strongly agreed and 29.17% agreed).

**Table 2:** Attitudes towards DF among healthcare workers in Somalia

Attitude level				Frequency *n* (%)	Mean ± SD
Negative Attitude (≤ 60%) (14–42 scores)				112 (29.1)	45.3 ± 4.2
Positive Attitude (> 60%) (43–70 scores)				272 (70.9)	
Total				384 (100)	

**Item**	**Strongly Agree** **n (%)**	**Agree** **n (%)**	**Neutral** **n (%)**	**Disagree** **n (%)**	**Strongly Disagree** **n (%)**
Dengue is a serious illness*	195 (50.78)	98 (25.5)	45 (11.72)	35 (9.11)	11 (2.86)
Dengue is a type of disease that cannot be prevented*	33 (8.59)	50 (13.02)	96 (25.00)	133 (34.64)	72 (18.75)
Everyone is at risk from dengue	92 (23.96)	143 (37.24)	97 (25.26)	42 (10.94)	10 (2.60)
Children are especially susceptible to dengue	101 (26.30)	133 (34.64)	124 (32.29)	18 (4.69%)	8 (2.08)
Dengue can be repeated (multiple infections)	81 (21.09)	117 (30.47)	144 (37.50)	33 (8.59)	9 (2.34)
Early stages of dengue are fully treatable*	62 (16.15)	88 (22.92)	126 (32.81)	68 (17.71)	39 (10.16)
Disposal of mosquito larvae is a waste of time and troublesome*	72 (18.75)	35 (9.11)	96 (25.00)	100 (26.04)	81 (21.09)
Killing mosquitoes carrying dengue is the only way to control or prevent infection*	146 (38.02)	123 (32.03)	76 (19.79)	35 (9.11)	4 (1.04)
I can play an important part in preventing dengue	160 (41.67)	173 (45.05)	43 (11.20)	5 (1.30)	3 (0.78)
Eradicating mosquito breeding grounds is the responsibility of health professionals and volunteers only*	97 (25.26)	133 (34.64)	81 (21.09)	56 (14.58)	17 (4.43)
Breeding grounds should be monitored yearly	156 (40.62)	112 (29.17)	90 (23.44)	24 (6.25)	2 (0.52)
Fogging can prevent mosquito breeding completely	95 (24.74)	112 (29.17)	123 (32.03)	51 (13.28)	3 (0.78)
Would see a doctor if having dengue signs	149 (38.80)	185 (48.18)	40 (10.42)	8 (2.08)	2 (0.52)
Health professionals are not required to inspect residential properties*	100 (26.04)	110 (28.65)	105 (27.34)	60 (15.62)	9 (2.34)

*Reverse statements were scored on a 5-point Likert scale, ranging from “strongly disagree” to “strongly agree”

### Practice assessment

Practice assessment revealed concerning patterns, with 61.6% demonstrating poor practices (mean score 11.7 ± 4.7) (Table 3). Water management practices showed variable compliance: 69.53% regularly closed water containers, and only 37.24% used proper water treatment methods. Vector control practices revealed implementation gaps: 65.10% were disposed of stagnant water, but only 35.42% were actively checked for mosquito larvae. Personal protection measures, including mosquito net usage (33.07%), protective clothing (32.29%), and mosquito coils (24.48%), showed particularly low adoption rates. Healthcare-seeking behaviour was the strongest practice category, with 87.50% reporting immediate hospital visits and 84.38% using appropriate antipyretic management.

**Table 3:** Practices towards DF among healthcare workers in Somalia

Practice level		Frequency *n* (%)	Mean ± SD
Poor Practice (≤ 60%) (0–14 scores)		236 (61.6)	11.7 ± 4.7
Good Practice (> 60%) (14–23 scores)		148 (38.5)	
Total		384 (100)	

**Category**	**Item**		**Yes n (%)**
Water Management	Water containers/wells are covered		192 (50.00)
	Close water containers after use		267 (69.53)
	Check stored water condition		207 (53.91)
	Use abate in water reservoir		143 (37.24)
Breeding Site Control	Check for mosquito larvae		136 (35.42)
	Dispose of stagnant water		250 (65.10)
	Check residence for breeding sites		130 (33.85)
	Remove water-collecting containers		239 (62.24)
	Clean gutters during rainy season		137 (35.68)
	Sleep under mosquito nets		127 (33.07)
	Have window screens/nets		245 (63.80)
	Use mosquito coils at night		94 (24.48)
	Use mosquito sprays at night		166 (43.23)
	Wear long-sleeved clothing		124 (32.29)
Community Engagement	Participate in prevention campaigns		116 (30.21)
	Consider eradication shared responsibility		298 (77.60)
	Allow authorities prevention activities		251 (65.36)
Healthcare Seeking	Immediate hospital visit		336 (87.50)
	Visit general practitioner		242 (63.02)
	Call ambulance		140 (36.46)
Self-Management	Wait to see if symptoms improve		243 (63.28)
	Take antipyretics and monitor		324 (84.38)
	Use traditional/herbal remedies		86 (22.40)

### Demographic variations in KAP score

Demographic variations in KAP score analysis revealed demographic patterns in KAP scores (Table 4). Age-related analysis showed that knowledge levels peaked in the 36–45 age group (41.2%), whereas positive attitudes peaked in the > 46 group (80%). However, good practices towards dengue were the lowest in this age group (40%). Gender analysis showed minimal variation across all domains, with females showing slightly higher knowledge levels (35.1% vs. 31.9%). Females also showed somewhat more positive attitudes than men. Marital status analysis revealed that divorced and widowed individuals had higher knowledge levels (60.0%) than married (34.4%) or single (30.2%) participants. The educational impact revealed that bachelor’s degree holders had higher knowledge levels (35.3%) than diploma holders (6.7%). Professional variation showed that clinical officers and dentists had the highest knowledge levels (50% and 40%, respectively), whereas nutritionists were concerned about deficiencies (0%). Clinical officers also had the best attitudes and practices, followed by dentists (Table 4). Regional analysis demonstrated disparities, with Puntland and Southwest regions showing notably higher knowledge levels (61.5% and 63.6%, respectively) than the other regions. The Puntland and Southwest regions also demonstrated better attitude and practice scores than other regions.

**Table 4:** Analysis of demographic variabilities (Knowledge, attitude, and Practice) among healthcare workers in Somalia

Variables	Knowledge		Attitude		Practice	
	Good	Poor	Positive	Negative	Good	Poor
Age						
<25	33 (27.5)	87 (72.5)	82 (68.3)	38 (31.7)	44 (36.7)	76 (63.3)
26–35	72 (35.8)	130 (64.2)	145 (71.8)	57 (28.2)	78 (38.6)	124 (61.4)
36–45	21 (41.2)	31 (58.8)	37 (71.2)	15 (28.8)	22 (42.3)	30 (57.7)
>46	2 (20.0)	8 (80.0)	8 (80.0)	2 (20.0)	4 (40.0)	6 (60.0)
Gender						
Female	61 (35.1)	113 (64.9)	123 (70.7)	51 (29.3)	66 (37.9)	108 (62.1)
Male	67 (31.9)	143 (68.1)	149 (71.0)	61 (29.0)	82 (39.0)	128 (61.0)
Marital status						
Divorced	6 (60.0)	4 (40.0)	7 (70.0)	3 (30.0)	4 (40.0)	6 (60.0)
Married	62 (34.4)	118 (65.6)	127 (70.6)	53 (29.4)	69 (38.3)	111 (61.7)
Single	57 (30.2)	132 (69.8)	134 (70.9)	55 (29.1)	73 (38.6)	116 (61.4)
Widow	3 (60.0)	2 (40.0)	4 (80.0)	1 (20.0)	2 (40.0)	3 (60.0)
Highest Education Level						
Bachelor	91 (35.3)	167 (64.7)	182 (70.5)	76 (29.5)	98 (38.0)	160 (62.0)
Certificate	1 (16.7)	5 (83.3)	5 (83.3)	1 (16.7)	2 (33.3)	4 (66.7)
Diploma	1 (6.7)	14 (93.3)	11 (73.3)	4 (26.7)	6 (40.0)	9 (60.0)
Postgraduate	35 (33.3)	70 (66.7)	74 (70.5)	31 (29.5)	42 (40.0)	63 (60.0)
Profession						
Clinical officer	1 (50.0)	1 (50.0)	2 (100.0)	0 (0.0)	1 (50.0)	1 (50.0)
Community health worker	1 (20.0)	4 (80.0)	4 (80.0)	1 (20.0)	2 (40.0)	3 (60.0)
Dentist	2 (40.0)	3 (60.0)	4 (80.0)	1 (20.0)	2 (40.0)	3 (60.0)
Lab tech	22 (37.9)	36 (62.1)	41 (70.7)	17 (29.3)	24 (41.4)	34 (58.6)
Medical doctor	60 (35.7)	108 (64.3)	118 (70.2)	50 (29.8)	65 (38.7)	103 (61.3)
Nutritionist	0 (0.0)	8 (100.0)	6 (75.0)	2 (25.0)	3 (37.5)	5 (62.5)
Pharmacist	3 (37.5)	5 (62.5)	6 (75.0)	2 (25.0)	3 (37.5)	5 (62.5)
Public health official	15 (36.6)	26 (63.4)	29 (70.7)	12 (29.3)	17 (41.5)	24 (58.5)
Midwifery	3 (15.8)	16 (84.2)	13 (68.4)	6 (31.6)	7 (36.8)	12 (63.2)
Nursing	21 (30.0)	49 (70.0)	49 (70.0)	21 (30.0)	28 (40.0)	42 (60.0)
Region						
Banadir	89 (32.2)	187 (67.8)	194 (70.3)	82 (29.7)	106 (38.4)	170 (61.6)
Galmudug	14 (31.1)	31 (68.9)	32 (71.1)	13 (28.9)	18 (40.0)	27 (60.0)
Hirshabele	5 (33.3)	10 (66.7)	11 (73.3)	4 (26.7)	6 (40.0)	9 (60.0)
Jubaland	3 (15.0)	17 (85.0)	14 (70.0)	6 (30.0)	8 (40.0)	12 (60.0)
Puntland	8 (61.5)	5 (38.5)	9 (69.2)	4 (30.8)	5 (38.5)	8 (61.5)
Somaliland	2 (50.0)	2 (50.0)	4 (100.0)	0 (0.0)	1 (25.0)	3 (75.0)
Southwest	7 (63.6)	4 (36.4)	8 (72.7)	3 (27.3)	4 (36.4)	7 (63.6)

### Correlation analysis

**Fig. 2:**
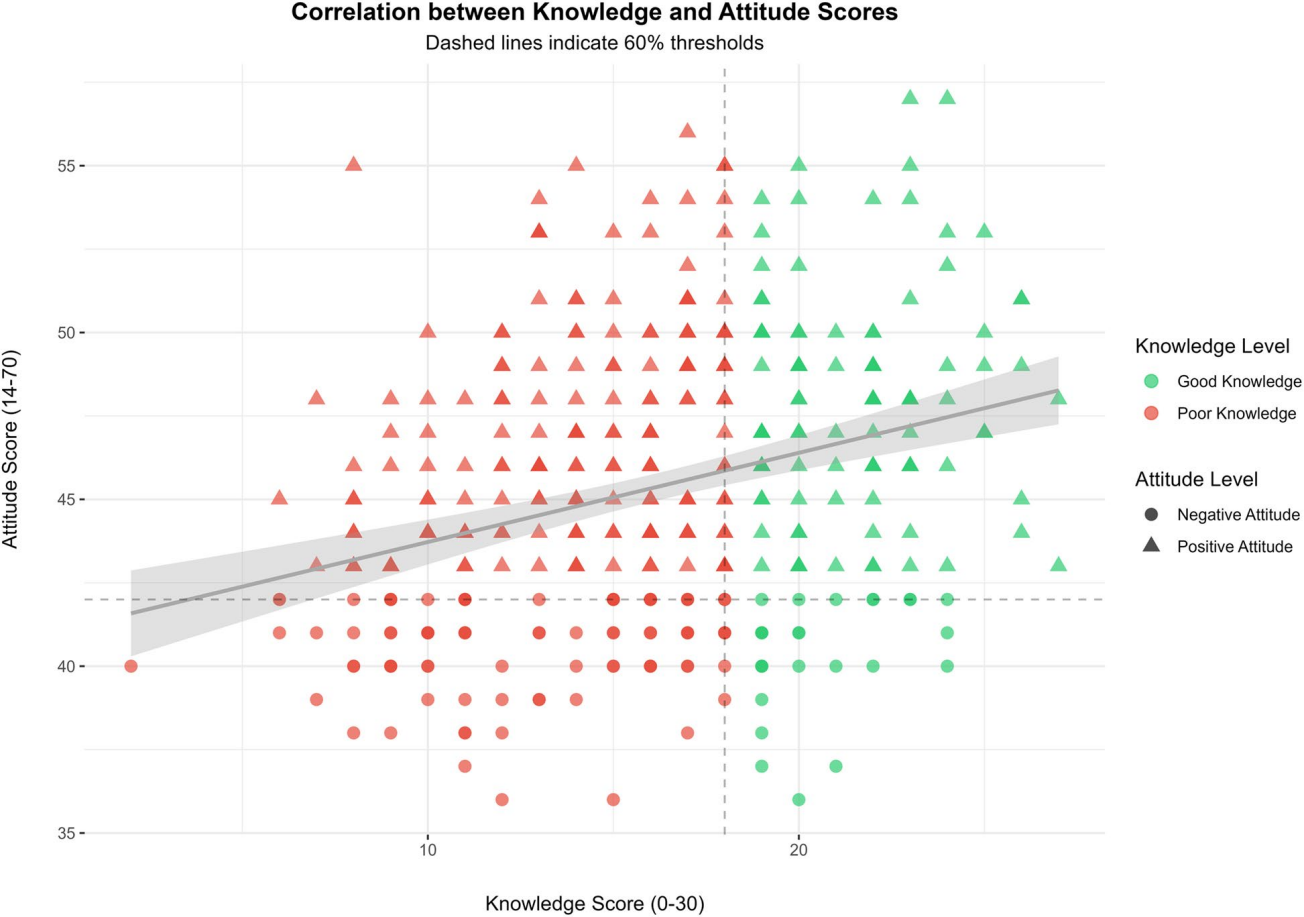
Correlation between Knowledge and Attitude Scores. Each point represents a participant. Colour shows knowledge level (green = good, red = poor), and shape shows attitude (triangle = positive, circle = negative). Dashed lines indicate 60% thresholds used to categorise scores: knowledge > 18/30 is considered good, and attitude > 42/70 is considered positive. A weak positive correlation is observed between the two scores

Statistical analysis revealed significant positive correlations between all the KAP components (*p* < 0.001), as assessed using Pearson’s correlation. Knowledge showed a moderate correlation with practices (*r* = 0.49) and a weak correlation with attitudes (*r* = 0.30) (Fig. 2), whereas attitudes and practices demonstrated a weak correlation (*r* = 0.25). These correlations suggest that knowledge improvement could positively impact attitudes and practices, although the relationship strength varied (Table 5). The stronger knowledge-practice correlation compared to the knowledge-attitude correlation suggests that knowledge may be more closely linked to behaviour than attitudes, indicating the importance of practical education in improving dengue management practices.

**Table 5:** Pearson correlation analysis of knowledge, attitude, and practice scores among healthcare workers in Somalia

Variable	Knowledge	*P* value	Practice	*P* value	Attitude	*P* value
Knowledge	1.00	-	0.49	< 0.001*	0.30	< 0.001*
Practice	0.49	< 0.001*	1.00	-	0.25	< 0.001*
Attitude	0.30	< 0.001*	0.25	< 0.001*	1.00	-

* Indicate statistical significance (*p* < 0.05)

## Discussion

This study assessed the level of knowledge about DF, attitudes towards DFDF, and practice among healthcare workers in Somalia. It provided critical insights into the general overview of Somalia to enhance decision-making and DF surveillance in the country. The need for this investigation was further justified considering the recent sporadic outbreaks of DF in different regions of Somalia [3, 8].

### Participants sociodemographic characteristics

In this study, we found that the healthcare workforce in Somalia is relatively young, with over 80% of the participants aged below 35 years, indicating a nascent healthcare system. This ndicates the growth of Somali healthcare after most experienced healthcare workers migrated due to the collapse of the healthcare service during the protracted conflict. Regarding gender distribution, the results showed moderate male predominance, while nearly half of the participants were single, reflecting a workforce likely in early career stages.

Educational qualifications revealed a well-educated workforce, with the majority holding bachelor’s degrees, although the relatively low proportion of postgraduate qualifications (27.3%) may indicate room for advanced training. These findings are consistent with findings from regions affected by instability and conflict, including the DR Congo [17]. However, the age range is slightly lower than the 31–45 years reported in Chad [18].

The study also revealed notable variations in the KAP scores based on age, education, and professional background. Knowledge was highest among those aged 36–45 years and bachelor’s degree holders, suggesting that experience and education positively impacted knowledge levels. Clinical officers and dentists demonstrated superior knowledge, attitudes, and practices, whereas nutritionists and midwives had lower scores, indicating potential training gaps. This finding is congruent with a similar study in Iran, where education level and work experience were identified as factors associated with good knowledge, attitude and practice among healthcare workers [19].

Regional disparities are stark, with over 70% of participants concentrated in Banadir, reflecting the uneven distribution of healthcare resources. The uneven distribution of the health workforce can be attributed to insecurity challenges in rural areas a the appeal of the booming urban life and better livelihood in the Banadir region, where the central government is located. This centralisation may limit access to trained professionals in other regions, highlighting the need for strategies to attract healthcare workers to underserved areas. Analysis of variabilities based on regions showed that Puntland and Southwest regions had higher KAP scores, suggesting that localised factors such as public health campaigns or infrastructure may have influenced these outcomes [3].

### Participants knowledge assessment

The study identified significant gaps in knowledge about DF, with 66.66% of the participants classified as having poor knowledge. While there is a strong recognition of common symptoms (e.g., fever, chills, and headache), awareness of critical warning signs such as bleeding and pain behind the eyes remains alarmingly low. These results are similar to those of Southeastern Iran, where poor knowledge and low education levels are prevalent among healthcare workers [19]. In neighbouring Sudan, an investigation among medical students revealed 58% good knowledge, while a similar study in Tanzania showed that 53.8% of healthcare workers have good knowledge [20, 21]. Knowledge about disease transmission was moderate, with 70.05% identifying mosquitoes as the vector, but only 17.19% correctly recognising peak activity times for Aedes mosquitoes. the recent sporadic outbreaks of DF in Somalia, the disease rarely occurred, which may explain the poor knowledge and awareness about the cause and transmission pattern of the disease. Moreover, prolonged conflict undermines healthcare workers’ disease knowledge by disrupting health systems, limiting training opportunities, and causing stress and burnout. This weakens care quality and heightens disease transmission risks in communities [22, 23]. This suggests a need for targeted training that focuses on the critical aspects of disease management. These findings underscore the importance of improving healthcare understanding of vector behaviour and warning signs, pivotal in early diagnosis and effective intervention.

### Participants’ attitudes and practice evaluation

Most participants (70.9%) exhibited positive attitudes, recognising their role as healthcare workers in prevention and the seriousness of DF. This finding agrees with results elsewhere, including Sudan (74.1%), Northern Iran (81%), and Eastern Ethiopia (52%) [20, 24, 25]. However, misconceptions were prevalent, with many believing mosquito elimination is the sole prevention method, and that eradication responsibilities lie primarily with health professionals. Although mosquito control is critical, comprehensive dengue prevention also involves community engagement, environmental management, early diagnosis, and public awareness campaigns. Innovative vaccine candidates have provided evidence of promising dengue prevention measures [4, 5]. Strategies such as removing breeding grounds, using personal protective measures, and improving access to healthcare are equally important. These misconceptions highlight the need to foster community engagement attitudes, as effective dengue control relies on collective responsibility. As earlier reported, some of the factors that contribute to these misconceptions as well as the poor attitudes of healthcare workers toward dengue include limited knowledge, lack of ongoing training, high workload, resource shortages, and low perceived risk of the disease, which are all factors abound in humanitarian crisis affected countries.

However, the poor practices were evident in 61.6% of participants, particularly in vector control and personal protection measures. This was also the case in Sudan, where less than half of the healthcare workers studied demonstrated good preventive practices [20]. This study also found that only 35.42% actively checked for mosquito larvae, and personal protective behaviours, such as mosquito net usage (33.07%) and wearing protective clothing (32.29%), were low. Attitudes and practices are interconnected; however, positive attitudes toward prevention do not always translate into effective practices because of gaps in knowledge and misconceptions. Some potential barriers to good practices that may account for the poor practices observed could be systemic barriers, such as low awareness and education, inadequate resources, and underestimation of community participation in prevention efforts [26]. Low level of practice towards DF prevention was reported to be associated with location of healthcare facility, with facilities located in rural areas more common. However, this study was conducted in the capital city of Mogadishu, an urban settlement. This might be due to one-third of our study areas being rural health centres where cases of DF were unusual. Another possible reason could be the lack of diagnostic testing.30 No published study has explicitly evaluatedthe practice level of HCPs towards DF prevention, limiting our comparison.

Notwithstanding, urban healthcare workers may show poor attitudes and practices toward dengue control due to limited knowledge, insufficient training, lack of resources, and challenges posed by overcrowding, inadequate sanitation, and abundant mosquito breeding sites [27]. These findings suggest gaps in translating knowledge and attitudes into effective practice. Nonetheless, healthcare-seeking behaviours were strong, with high rates of immediate hospital visits and appropriate antipyretic use.

In general, significant positive correlations were observed among all KAP components, with knowledge showing a stronger correlation with practices than attitudes. This indicates that improving knowledge may directly influence behaviour more than perceptions, emphasising the importance of practical hands-on education in training programs. Furthermore, knowledge plays a pivotal role in DF management by directly influencing practices and indirectly shaping attitudes [28–30]. Addressing knowledge gaps among healthcare professionals is crucial for enhancing their role in prevention and control. By fostering a well-informed workforce, the healthcare system can better mitigate the dengue burden, reduce transmission, and improve patient outcomes.

## Conclusion

Despite the generally positive attitudes observed, this study highlights substantial gaps in knowledge and practices related to dengue fever (DF) among healthcare professionals in Somalia. A significant proportion of respondents (66.66%) demonstrated poor knowledge, and 61.6% reported poor preventive practices, underscoring the need for targeted interventions. Knowledge was found to have a statistically significant moderate correlation with practice (*r* = 0.49, *p* < 0.001) and a weak correlation with attitude (*r* = 0.30, *p* < 0.001), while attitude and practice were also weakly correlated (*r* = 0.25, *p* < 0.001). These findings suggest that strengthening knowledge among healthcare workers will likely yield improvements in attitudes and practices toward DF prevention and control.

To address these gaps, strategic efforts should prioritise comprehensive training programs focusing on critical knowledge domains, including recognition of warning signs, understanding vector behaviour, and the importance of hydration and management protocols. Furthermore, enhancing community engagement through awareness campaigns is crucial to dispel misconceptions and promote collective responsibility beyond health professionals. Behavioural interventions encouraging personal protective measures and active participation in vector control activities are also recommended to translate positive attitudes into effective preventive practices. Strengthening healthcare workers’ knowledge base, particularly in regions outside Banadir, will be key to reducing the burden of DF and improving public health outcomes in Somalia.

### Study limitation

Our cross-sectional study approach would not have allowed us to deduce a causal relationship. Secondly, because we relied solely on self-reported data collection, we could not rule out potential recall or social desirability bias, which could affect the accuracy and authentic expression of the participants’ understanding of DF. Notwithstanding these limitations, the findings of this study provide valuable insights that can help formulate targeted interventions and implement preventive strategies against DF.

## Data Availability

Data is provided within the manuscript or supplementary information files.

## Abbreviations

DF: Dengue fever
HCW: Healthcare workers
KAP: Knowledge, Attitude and Practice
AMISOM: African Union Mission in Somalia
